# Recurrent Cellulitis in a Patient with Papillomatosis Cutis Lymphostatica

**DOI:** 10.5811/cpcem.2017.10.36031

**Published:** 2018-01-09

**Authors:** Stella Pak, John-Phillip Markovic, Yan Yatsynovich, Ethan Tope, Damian Valencia

**Affiliations:** * Kettering Medical Center, Department of Medicine, Kettering, Ohio; † Wright State University, Boonshoft School of Medicine, Dayton, Ohio

## CASE PRESENTATION

A 61-year-old female with a history significant for polycystic ovarian syndrome complicated by splenic cysts status-post splenectomy and chronic lymphedema presented to the hospital with cellulitis involving both lower extremities. In the prior eight months, she had six episodes of cellulitis caused by group B *Streptococcus* involving her lower extremities. She was hospitalized, and blood cultures grew out group B *Streptococcus*. She received treatment with intravenous levofloxacin and vancomycin and demonstrated clinical improvement. However, careful inspection of the area of cellulitis on her lower extremities revealed papillary lesions consistent with a condition known as papillomatosis cutis lymphostatica (Image). For this condition, she was treated with compression stocking and amoxicillin 500 mg four times daily in the outpatient setting.

## DIAGNOSIS

Papillomatosis cutis lymphostatica is a rare complication of primary or secondary lymphedema and has limited treatment options.^1^ It increases the risk of infection by causing mechanical tearing of the papules and subsequent breakdown of the skin barrier, which provides a portal of entry for bacterial invasion. Use of compression stockings is the cornerstone of conservative management.^1^, ^2^ Vitamin A derivatives, such as acitretin, have shown therapeutic efficacy in several cases. The postulated therapeutic mechanism by which vitamin A derivatives work includes interference with epidermal proliferation and inflammation by causing increased cell turnover through alteration of gene expression.^3^ Topical ointments, including 5% salicylic acid, and surgical interventions are other potential treatment options.^4^ This patient was managed with conservative therapy since she was a poor surgical candidate. However, early recognition of papillomatosis cutis lymphostatica is crucial to preventing recurrent infections. To the best of our knowledge, this is the first case of papillomatosis cutis lymphostatica complicated with bacteremia.

CPC-EM Capsule

What do we already know about this clinical entity?

Papillomatosis cutis lymphostatica is a rare complication of chronic lymphedema associated with recurrent cellulitis.

What is the major impact of the image(s)?

This is the first case of papillomatosis cutis lymphostatica reported in the emergency medicine literature.

How might this improve emergency medicine practice?

This image will help clinicians recognize and treat papillomatosis cutis lymphostatica. In the ED, recognition of this rare complication would allow clinicians to have an appropriately high level of suspicion for cellulitis.

## Figures and Tables

**Image:**
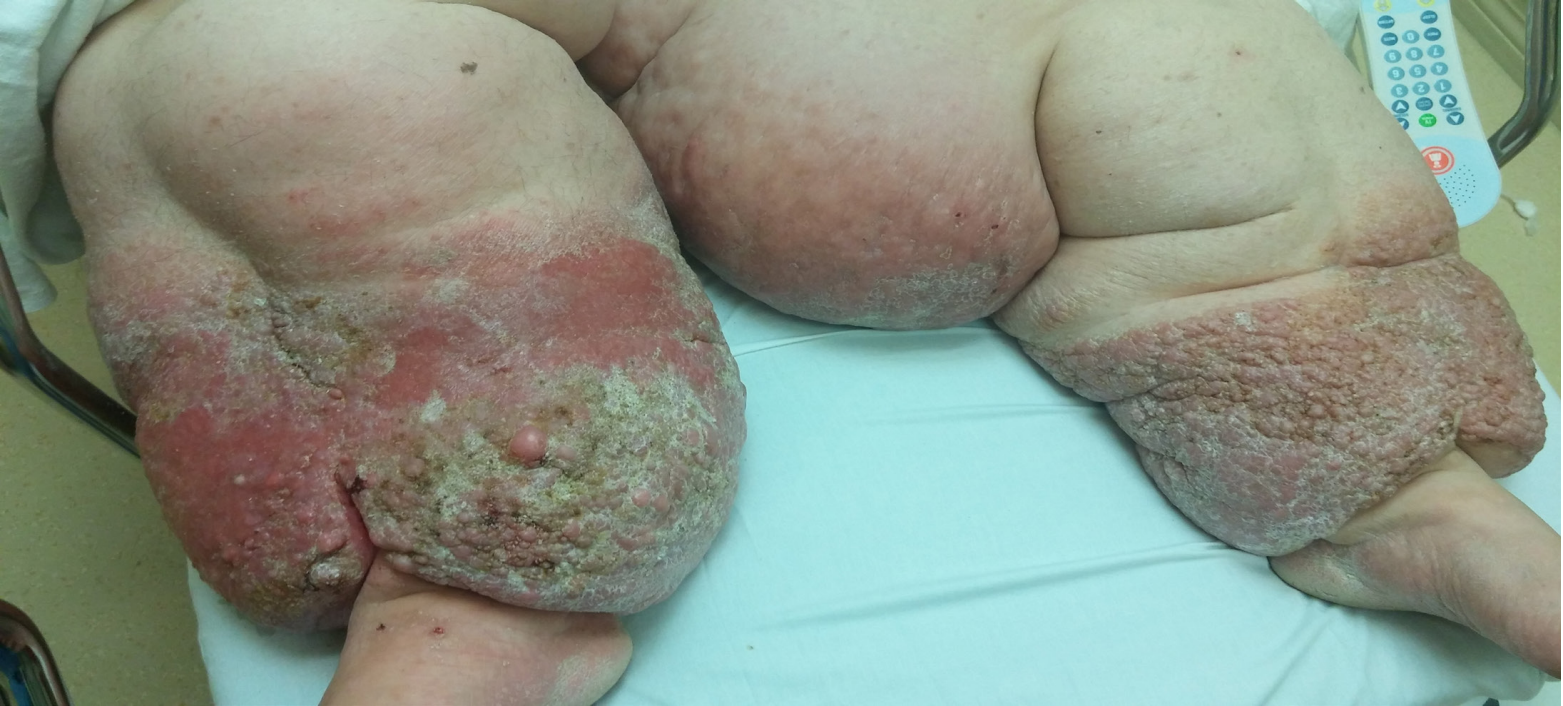
Multiple papular lesions on both legs indicate papillomatosis cutis lymphostatica, a rare complication of lymphedema
